# On Instinct and Reason; or, the Intellectual Difference between Man and Animals

**Published:** 1862-01

**Authors:** James Quilter Rumball


### Art. II.—ON INSTINCT AND REASON; OR, THE INTELLECTUAL DIFFERENCE BETWEEN MAN AND ANIMALS.

By James Quilter Rumball, M.R.C.S., etc.

In a former essay upon this subject* I endeavoured to show that all preceding writers had failed to establish any line of demarcation between the intellectual faculties of man and animals, and that their denial of reason to the brute was unsupported either by argument or fact. I proved that animals have reason differing only in degree, not in kind, from that of man, and that the only intellectual difference consists in the possession by man of articulate speech, which no other animal has.

I traced up the formation of brain from its lowest to its highest development, and established the two following propositions:—

1st. The brain is the organ of the mind.

2nd. As brain so mind.

Before entering upon the second portion of this inquiry, viz., "*The Moral difference between Animals and Man,*" it is necessary to apply my doctrine to an elucidation of some other intellectual results, such as "understanding, judgment, and memory;" and if we find that these terms no longer express indefinite ideas, but have each of them a clear and practical meaning, we shall obtain a strong *a posteriori* confirmation of the soundness of the data whence they spring. Much of our misconception and most of our disputes arise from the abuse of terms, or, at all events, from an extreme laxity in their use. If I refer to Johnson for the meaning of the word perception, I find it described as "the power of perceiving; knowledge; consciousness; notion; idea; feeling;" and he quotes Watts, Bentley, and Hale in support or these definitions; whilst Lord Bacon attributes perception to plants, and even to mountains. What sort of pyschological philosophy must that be which permits or is based upon terms thus evasively applied, may be gathered from the wild theories it has produced and the useless results obtained. In my former paper, I have restricted the word to what appears to be its essential signification, namely, *The cognition by the mind of external objects through the medium of the senses*, or, in other words, *the means by which we become acquainted with the physical properties of bodies*. This includes all impressions coming from without,

---

* See *Journal of Psychological Medicine*, vol. iv. p. 392.

and is embodied in the Aristotelian dictum, "Nihil est in mente quod non fuit primum in sensu." But the proposition is only true when restricted to our knowledge; for although we have no innate notions or ideas, all our *feelings* are born with us, and are merely developed into activity as we grow. This, however, will be treated of at length when we come to consider the feelings. Such, however, is the force of habit and the difficulty of coining new words, that though I may be compelled to use the term perception when speaking of moral cognition, yet do I protest against this double application of what should never be employed but in a single sense.

The word "reflection" apart from optics (wherein it seems used definitely enough), is also applied to mental operations, and if confined to the definition "Thought thrown back upon the past, or the absent, or itself," I know not if much mischief would ensue. It is used in one or other of these senses by Dryden, Locke, and others, but when the latter declares it to be the "Action of the mind upon itself," he includes all passions and their results; the more violent and the more lasting in exact proportion as they are disconnected from reflection.

I accept Johnson's definition of the word "judgment." He says it is "The power of discerning the relations between one term, or one proposition, and another," and I think it may be illustrated as follows.

Suppose a man blind from his birth could be placed, like Mahomet's tomb, midway 'twixt earth and heaven, with no object visible but "the blue above and the blue below," interminable, shadeless, shapeless; and that he could then and there discharge the "drop serene," and suddenly and perfectly obtain his sight.

It is evident that the simple perception of colour would be the only visual one called into existence; and as we will also demand that he shall never have had the word colour named to him, or have formed any conception of it, so will he have no previous mental perceptions with which to compare what he now sees, nor will he have any data whereon to exercise his reason. To him it will, therefore, be a simple perception without antecedent or relation: he consequently forms no judgment upon it.

Suppose another man to be placed in the same spot; his vision having been perfect from his birth. He at once exclaims that a blue colour surrounds him. Comparing it with past perceptions he names its shade, its apparent and probable extent, and as many of its other relations as to him are known. He judges, but his judgment is imperfect.

Suppose a wind to arise, and the orbs of day and night to appear, the earth still distant, but near enough to delight him with its infinite variety, he now begins to compare distances, but never having been so placed till now, his perception of perspective is very inaccurate. He judges better, but still ill.

Land him on the mountain-top, and all the elements necessary to right judgment are present. The blue above is the sky he had so often admired; intangible as he had lately experienced, boundless as it seemed, involving the earth as well as stars, and not the well-defined arch he hitherto had thought it: so that his elevation has improved his judgment, but not perfected it. Chemical and optical knowledge may add further to his stock of facts and inferences, but so long as essences and laws, moral or physical, are abstract principles and not appreciable entities, so long will human judgment necessarily remain imperfect. Still simple perception is not judgment. The blind man saw, but judged not; and till comparisons are made no judgment can be formed.

All those metaphysicians, then, have erred who consider judgment a fundamental power of the mind. Gall was equally wrong in calling it the third degree of activity of every fundamental faculty; and Spurzheim was as little accurate when he declared that "there are as many kinds of judgment as there are perceptive faculties." But he himself is dissatisfied with this, and goes on immediately to say, "The regular and perfect manifestation of the (two ?) reflective powers, however, examining the relations of all the intellectual and affective faculties to their respective objects, and the relations of the various powers among themselves, particularly deserves the name of judgment!"* It is necessary, then, to right judgment that everything belonging to the matter before the reason should be present to it, and that the reflective powers should be perfect, but as neither of these postulates can be granted, so perfect judgment can never be arrived at by mortal man. True! the reflective faculties only judge; perceptions are the tools with which they work; but perceptions do not judge, and a man's judgment will be more or less correct as his perceptions and consequent reflections are so.

Understanding comprehends more than judgment, as will presently be shown, but both terms have been ever used in a loose and unsatisfactory manner. If Spurzheim be not quite correct in his definition, a very little alteration of it will make him so. If we say that judgment consists in a combination of facts and arguments, and will be more or less accurate as they are comprehensive and true, or in other words, that "the result of our reflections upon our perceptions constitutes judgment," we shall have rendered his definition more precise, and, as I believe, more

---

* *Philosophical Principles*, p. 27.

true. The argument derived from the preceding remarks flows from all possible perceptions, intellectual or moral. Whether I feel love or anger, am delighted with painting or music, examine a question, political or religious, mechanical or metaphysical, some, and only some, of my mental powers are engaged; but whatever may be the nature of my perceptions, whether physical, as of natural objects, or affective, as of moral or social sympathies, it is only when I have reasoned upon them that my judgment is formed. Many or few may be my perceptions, hasty and untrue my deductions from them, wit may jump to general conclusions unwarranted by the particulars, comparison may reject all analogies, and so leave out the larger data, causality may assign causes before effects are proved; all these errors will detract more or less from the value of the judgment when formed, but touch not the judgment itself; and though Spurzheim is near the mark if we confine his definition to perfect judgment, yet do I believe that as there can be no such a thing with man, it will be found more accurate to define judgment as "the conclusion arrived at by all the mental faculties engaged." Many judgments, therefore, are directly opposed to each other, may be formed upon the same subject, not only by different individuals, but by the same person, and "quot homines, tot sententiæ" is a philosophical truth.

But if the foregoing be true in respect to our judgment of physical existence, and especially of things exernal to ourselves, it is even more palpably so when we attempt to judge of our own emotions. They judge not, but our intellect judges of them. Feeling (until a better word be found) is perception; and not until we can analyse and correct the perception with previous ones, and look as well to cause and consequence, are we able to form any idea of the nature even of a feeling.

Thus men often think that they are acting under one feeling, when the world sees plainly that they are impelled by another; the alms that self-love attributes to benevolence may be but too palpably traced to vanity, and what we think love is ofttimes fancy, or worse. So difficult indeed is it to judge of our feelings, that some men never try.

We cannot investigate the nature of a feeble emotion, that vanishes before we can examine it, nor a powerful one, which blinds our intellect by its violence; and, as it will hereafter be shown that the lunatic is only mad because there is no internal gauge by which he can measure his emotions, and therefore he acts under excited ones, as though they were still healthy, so in passion, or even less excited states of feeling, we are apt to compare our emotions one with the other as if all were equally sound, and our judgment is incorrect accordingly. Thus the world always doubts the propriety of any counsel dictated by feeling; and a review of every man's life will be a strange one if it do not reveal to him that 'twere well had he always done so too.

Judgment, then, is but conviction more or less correct; and conviction is but another word for belief—not that which men profess, but that which they inwardly acknowledge, except, perhaps, that these terms do express degrees of judgment. "I believe so," implies less perfect judgment than "I am convinced of it." "I think so, am certain of it, cannot be deceived," are all so many expressions indicating more or less reliance placed upon our judgment by ourselves when considering the data on which it is built.

That animals perceive and judge of external objects requires no argument to prove; that they reason upon their own emotions is more than doubtful; they judge as man judges of things they can perceive, but there is no inward looking, no self-examination; and this constitutes one of the prerogatives of humanity.

Understanding may be called passive judgment. "I understand" implies "I comprehend," that is, I have formed a judgment of the relations existing between any given subjects, and have examined the results.

A friend is in a dilemma, he lays his case before me, I understand it. He asks me how to extricate himself, and after mature examination of all probable means and consequences, I give him my judgment. I understand chemistry; I judge of its value to the arts and to its professors. I understand the world; I judge best how to go through it. My understanding has been built up upon perceptions reasoned upon, but passes not beyond them. It takes no account of modes of action incidental to them; I may understand how to do a thing, and why I do it; but I judge of the best time and manner as of the propriety or not of doing it. I can understand why I so judge, and judge why I so understand. The one, then, is the minor and the other the major of the same proposition. We cannot understand until we have reasoned, but the process of reasoning is not understanding any more than it is judgment.

It was abundantly shown in the essay on Instinct and Reason that animals perceive and reason, that they therefore understand and judge, and that they differ only in degree, not in kind, with respect to both, from the wisest among us. That animals have memory is so evident that it were waste of time to argue the point. The only distinction between them and man seems to be that they have the better memory of the two. Ulysses' dog was the only one who remembered him after his long wanderings, and horses seem never to forget a place they once have seen. But it may not be out of place here to attempt a definition of memory itself, seeing that two opposite opinions have been held by philosophers about it: one set declaring it to be a primitive faculty of the mind, the other that it is no more a primitive faculty than is imagination or wit!!

Phrenology rightly interpreted teaches that it is *the highest power of any perceptive faculty.* To see is one thing, to look another, to look long and steadfastly with good eyes and brain is a third, and memory is good or bad, as these latter conditions are fulfilled. The feelings have no memory. I can remember that the wine was good or bad, that I was happy or miserable, excited or depressed, but I cannot feel either the one or the other unless I drink similar wine, or am placed again in similar positions. But this has been already discussed, and we have now to consider what it is that constitutes the moral difference between other animals and man. And as this is a subject perfectly ignored by all writers preceding Dr. Gall, so to him is due the honour of having not only broached the question but solved it.

In prosecuting this subject, it seems necessary to advert very briefly to the origin of man. For although to Christians the Bible sufficiently demonstrates him to have descended from one pair, yet to those who know not or believe not the inspired book, other facts and other arguments must be adduced. The psychologist must address himself to all people, and to all times, and there must be no question raised as to the authority he quotes. If man has arisen from many stocks, then may the doctrine of Monboddo be in part correct, men may be merely tailless monkeys, and the gorilla will be one of us: but man—

> "Distinguished link in being's endless chain,
> Midway from nothing to the Deity,"

first link in creation, last of divinity, in both, of both, will be found to possess in common, attributes assignable to him alone, and his derivation from one stock is, I think, easily demonstrable. For however, and wherever in the beginning man was created and placed, we find that now, although he lives in every zone, and maintains his supremacy in all, he does so at the expense of much of his original perfection; he has in all instances become identified with his altered habits, and offers corresponding indications of his locality; nations, provinces and districts, trades and professions, cities and families, stamp their offspring with a peculiar, a distinct, a never to be mistaken character.

And as like produces like, the constitutional tendencies of parents, their vigour or debility, sagacity or folly, descend as heirlooms to their children, thus fulfilling the law which has declared that "The sins of the father shall be visited upon the children unto the third and fourth generation."

If our bodies are strongly tenacious of habit, they are equally slow to acquire it. If it take years to establish a peculiar character in a nation or family, it will require years also of equally powerful operating causes to remove that peculiarity; and this is the true history of predisposition whether of mind or body. I know that this has been denied. Lawrence, in his Lectures on the natural history of Man, says, that climate produces only a temporary effect, which dies with the individual. That if a white man go into a tropical climate, and contract a colour as deep as the native there, his child will be white, and his child's child, and all succeeding generations so long as the world shall last; and he cites the Jews as examples of the truth. They are to be found in every climate under heaven, and are not altered one jot from the likeness and habits of their forefathers. But it should be remembered, that long before their dispersion they lived sheltered from the sun in house or tent, were clothed in soft raiment, and fed on the produce of an overflowing land, and even now are only to be met with in cities where the art of man opposes and counteracts the influence of nature. But transpose a Jew into the wastes of Africa, and let him and his descendants hunt the beasts of the forest for their food, wear their fat as their only garment, have the bare earth for their bed, and the sky for their canopy, as the children of Ham did and do, and how many generations would it take to make him as black as Afric's darkest child? That the children of negroes are born white or pink coloured, as told us by Goldsmith and Pritchard, not only destroys the argument of Lawrence, but completely confirms the scriptural account of the primæval origin of us all, and proves also that the first man was white; for a black child from white parents has never yet astonished the world. The primitive blood then sometimes does appear and tell us whence we come.

However, Dr. Pritchard in his admirable book on the *Physical History of Mankind* considers it sufficiently evident that we do all proceed from one stock, and that climate, and occupation and habits transmitted from parent to child, constitute the differences observable in the human race.

It may be well to quote some of his arguments in support of the assertion.

The duration of life, of utero-gestation, and the internal organization, are universally similar, and have ever been so among all the races of men.

The progeny of the black and white breed; they are therefore not hybrid, for hybrids never breed.

"Negro children are white when born, but soon change." Occasionally the children of negroes remain white.

The differences of colour observable in the human species are equally prevalent among animals.

In Guinea, dogs and fowls (gallæ) are all black; in the Mysore, the same breed of sheep are red, white, and black ; the wild horses in the Pampas of South America are brown, the tamed ones of all colours ; xanthous and albino varieties spring frequently from melœna ;* the wild ass (onager) has a tuberculated skin, from which shagreen is made, but this is lost in the tamed animal.

In some hogs the hoof has become solid, in others five-cleft. Some fowls (as the Dorking) have five claws, some are without any rump, others with feathered legs, and some with their feathers turned the wrong way. In the Isle of Man the cats have no tails. The deviations from a common model in mankind, then, are less in degree than those which are found in many other species, and are analogous in kind as far as such analogy can be expected, and perhaps as conclusive as analogical reasoning can be upon this subject.

Let us then examine whether there is any visible connexion between mental and bodily constitutions thus produced, and whether the appearance of the one is any indication of the actual quality of the other.

That the outward forms of all animals do indicate their inward natures, is notorious to all. The form best adapted to their modes of life has been given to each. The lion, tiger, lynx, leopard, panther, cat, and indeed all the carnivoræ, are armed with great muscular strength in their jaws, necks, and limbs. They are furnished with sharp, curved, and cutting teeth, for tearing their prey, and an exquisite perfection of the senses of smelling and vision. Among birds the powerful crooked beak, enormous talons and eagle eye, are sufficiently indicative of certain habits, nor are fishes wanting in corresponding indiciæ. All the above have a savage character; in a state of nature are ferocious and courageous, and are incapable as well as unconscious of kindness.

Shall we say that here is no external evidence of internal faculties or propensities, or should we not, upon seeing a new species with such a conformation, conclude that the corresponding disposition was not wanting, and that it was equally allied to its class in character as in form ?

The light elegant form of deer, the antelope, hare, and others, is constituted for speed.

The more powerful among the former are armed with horns, and

---

* A man named Pearce, who lived in the Bull Ring, Birmingham, some twenty years ago, was a perfect Albino. No other member of his family (and they were many) was born so; and he was said to be affected in his mother's womb by a neighbouring gossip giving her a vivid description of an Albino exhibited at the fair.

in the rutting time are as bold and as ferocious as the lion himself, but as a rule this class is powerless for attack. Their safety consists in flight, courage in them would only invite destruction; they are therefore gifted with great timidity, and an extraordinary quickness of hearing, seeing, and smelling the approach of an enemy. Here again we have a strict connexion between the external and internal being.

Domestication considerably modifies the original character of some animals, but never entirely changes them. The bulldog is courageous, the spaniel fawning, the greyhound swift, and their forms at once proclaim their breed, but the type is ever present in them all. Those accustomed to horses, know that the full round forehead and ears wide set, indicate a good temper and high courage, whilst a hollow forehead and ears set narrowly on the head, denote timidity and vice.

We find then throughout the animal kingdom that,

*Form indicates character*, and that nature never gives power of any description without the propensity to use it. Animal physiognomy therefore, is easy of attainment and never deceives.

"We have now to inquire whether form, which denotes individual character in beast, is significant of similar characters in man." So says Lavater, and we cannot do better than follow his plan. But we shall find this a more difficult operation, as the mental faculties of man are more complex. "He has had given to him not one character or quality, but a world of qualities interwoven with, and obscuring each other; his mind consists of interminable combinations of a few simple powers, and the indications of these powers will be as numerous and obscure." In him too a continual struggle is going on between what he really feels, and the feeling he wishes to exhibit, which will create a confused manifestation, compounded of the emotion experienced and the effort to conceal it: men's faces are so marked with this constant warfare that hypocrisy becomes their prevailing character, as it is their all-prevailing propensity.

Some forms express so little, and have so little to express, that no determinate judgment can be formed of them. They are blanks wherein is seen nor good nor evil. "They die and make no sign." Much difficulty will also arise from our own limited powers of observation. Man judges of others by himself; "the best, the greatest, the most philosophical physiognomist is still but a man, a prejudiced man, and to judge perfectly, he must himself be perfect."

Nature is, however, always true to herself. She never errs nor deceives, and if we cannot always find her out, "the fault is not in the object but in the observer." There are, however, characters so marked, connexions so palpable, and consequences so inevitable, that "he who runs may read." The physiognomical evidences of wisdom or of folly, the slightest shades of some passions and the extremes of all, are written upon the faces and the forms of men as truly as beauty or deformity. "It cannot be too emphatically repeated that blind chance and arbitrary disorder constitute the philosophy of fools."

"Traversing the face of the earth and beginning at the north, we find in Lapland, and on the coast of Tartary, a race of men small of stature, singular of form, and with countenances as savage as their manners." The intellectual faculties of these northern nations are extremely feeble; they are bound up in the same icy fetters which equally forbid the growth of fruit or flower. Everything is degraded, deformed, desolate, and man is a type of all around him. These nations resemble each other in ugliness, size, colour of their hair (which is black), in their gross manners, inclinations, superstitions, cowardice, stupidity and indecency.

As we proceed eastward, the features of the tribes soften until we come to China, where we find a fat, well-proportioned race, more than half civilized, industrious and peaceful.

The people of Cashmere are renowned for their beauty; Buffon says that "no ugly face is to be found among them," and that "the men have good understanding, but bad education renders them vicious; nevertheless, they are civil, humane, and moderate."

The natives of Van Dieman's Land, New Holland, New Guinea, and some neighbouring islands, are even yet "hideous savages;" they are but one remove above a monkey, and want even his attachment to their females and their offspring. All writers describe them as amongst the most degraded of the human race.

The differences between the Irish, Scotch, English and French, are too familiar to require comment.

Tacitus attributes to the Greeks the same precipitate volatile gaiety; to the Britons the same cool, considerate, intellectual talent; to the Germans the same bold, virtuous, self-denying prudence, which are strong characteristics of the same nations at the present day.*

From the accounts of travellers it appears that there are as many varieties among the blacks as among the whites. On the coast of Guinea the natives are remarkably ugly; those of Sofala and Mozambique are handsome; those of Senegal the best informed; of Cape de Verd, well-made, their features soft and tempers mild.

The Phrenological Society of Edinburgh possesses sixteen

---

* The result would almost seem to justify the remark of Lavater, that "the morally best are the most beautiful; the morally worst the most deformed."

skulls of Hindoos that completely bear out the character of this race, which is kind and virtuous, but timid, cunning, and fond of metaphors. All the skulls obtained from Egyptian mummies present a European development:—the arts and sciences were cultivated among the ancient Egyptians as with us.

It has been said that all the members of a royal family in Africa, although black, possess Roman noses! The poet remarks a facial peculiarity in the Austrian;

"Why boasts the Austrian lip the Austrian line?"

Compare the Laplanders and the Greeks even of our day. The former are stunted in form, and brutes in intellect: "Scarce half made up, and that so lamely and unfashionably that the dogs bark at them."

The latter have descended from a nation of sages and heroes, who, when the world lay in savage darkness around them, formed a constellation of beautiful forms and bright minds, scattering rays of knowledge into all space and time, and realizing in their own forms their boldest conception of the Godhead.

Between twenty degrees of latitude and fifty-five, dwell the élite of mankind. In these climes the temperature is mild, food abundant, and the opportunities for bodily and mental exercise innumerable and uninterrupted. In the tropics a burning sun renders any exertion, either mental or bodily, distasteful and difficult; and in the frigid zones men spend half their time in seeking and storing food for the remaining half, which they pass in a state of almost torpid insensibility. Six months in the year do they lie buried in continued darkness coiled up in a cave, covered with moss or with fern, and only wake into a foraging existence when the sun revisits their inhospitable homes. The gradations between these two extremes are numerous and regular, as are the changes of climate and of the earth's face from the pole to the equator; nevertheless the perpetual intermixture of one variety with another now in progress—the commercial intercourse between far distant nations and the active dispersion of information in all, must materially confuse and soften down effects derivable from external influences, and draw more to one level the present widely-separated descendants of Adam.

However great the variety therefore observable in the human as in other races, there is no one so great as to indicate a different species or origin, and the genus homo may, for all ethnological purposes, be considered as one, and compared with other genera accordingly. That he is deteriorated and fallen is provable both by fact and argument.

He evidences no powers which, however extended, could create himself. It is impossible to consider him as having existed for ever; therefore he was created by some other power. Now in the beginning he was either created perfect man or he was not; if imperfect, then every improvement in his nature is a new creation. But the history of all living things proves that each class was formed upon a certain model, which is much departed from when subjected to local influences; that the breed degenerates under unfavourable circumstances, and that this degeneration may be arrested and the higher qualities restored by removing for several generations those deteriorating influences. But no one will contend for the possibility of eliciting attributes not inherent in the race; therefore a perfect animal must embody in all fulness the whole of the properties of the whole class. A perfect man must evidence all the powers of all men. No one mental power will be stronger or weaker than the others, just as a Venus or an Apollo exhibits no part of the body more beautiful than the rest. So must the perfect man be in his own person capable of all that the whole race can do.

Since Adam there has only been one of whom this might be predicated, and it is not irreverent to suppose that it was intended to show forth in his humanity an example of what man may be, in illustration of the doctrine which taught him what he ought to be. Now as no man is perfect, and as God created all things "good after their kind," man has fallen! how or when philosophy teacheth not, but the Bible does, and all we know of ourselves confirms the fact. Men differ from each other in what each has lost, not in anything any one has gained. The old Adam is in us all, but shivered and broken.

We have now to investigate the *moral nature of man*—and to Dr. Gall we owe the only clear and definite description of it we possess. I shall not go over the ground again I so wearily trod in my first essay, but briefly expound his doctrine which, if found to be true, upsets every preceding theory and constitutes, as I believe, a second revelation to man. The first trace of mind evidenced by the new-born animal would seem to be consciousness, which instantly excites volition; muscular motion ensues, and then hunger. The motions of the fœtus in utero are probably automatic, devoid of consciousness and certainly of hunger. Consciousness is excited through the medium of the senses, and in the child pain is the first evidence of it; in animals this pain is not so certain. Hunger arises from within, and is an innate instinctive propensity of so much importance that life itself depends upon it.

This desire is manifested by all animals, including man; it varies much in various classes; it is entirely independent of reason, and acts prior to it; neither the child nor the lamb is aware of the necessity for eating or drinking, nor can it create the desire; it is an instinct, and not an intellectual faculty, evidently depending for its manifestation on the brain or some portion of it, for if you divide the nerves proceeding thence to the stomach, hunger is destroyed; and as I have had 1200 professional opportunities of testing the organology of Gall, I unhesitatingly avow my perfect belief in his doctrine. However, it is sufficient for my present purpose to treat hunger psychologically, deferring all craniological discussion for the present.

Until Dr. Crook called the attention of phrenologists to the subject, it was thought that hunger and thirst were but simple modifications of the same instinct; and that alimentativeness, the term given to it by Gall and Spurzheim, and adopted by Combe, comprised all that we know of both. But when we find that the horse, the cow, the goat, and others, not only accurately distinguish, and invariably select those herbs only which are suited to their natures, and this without any teaching, but as constantly reject those which are unsuited to them, and this not by any instinct common to them all (for the cow will eat what the horse refuses, and so on through the whole of the herbivorous tribes); that carnivorous animals do not eat herbs, and that children instinctively reject some food which other children prefer, we must agree with him that gustativeness is a better term. But hence arises another difficulty: are hunger and thirst but modifications of the same faculty? If so, they should appear and disappear together. Whereas exactly the opposite is the fact. A horse will cease eating his corn until you give him drink; man's hunger gives way to his thirst; what excites the one has no effect upon the other. He who drinks most often eats least, and men cast away at sea, frequently die mad from thirst long after their hunger has left them. There are, therefore, two propensities common to man and brutes: the desire for food and the desire for drink, and, as it will hereafter be proved, there are two portions of the brain that manifest them. I retain both the terms.

Either faculty may be abused by man, but not by any other animal; gluttony is the abuse of alimentativeness, epicurism of gustativeness. If the instincts were one, the duck and the pig should be as dainty as they are voracious, and the ass as voracious as he is dainty, but this is not so. Again, the portions of brain manifesting them may be diseased. In bulimia, or depraved appetite, alimentativeness, in fever gustativeness, is affected.

There was a French prisoner at Portsmouth who was tested one day by his medical attendant; he ate sixteen pounds of food in twelve hours, six pounds of it consisting of soap and candles. Birds do not seem to possess any innate power of choosing proper food for themselves. The mother invariably feeds her offspring. The chicken will pick up gravel stones or anything, until its mother directs its choice by taking up that which is proper, clucking her little ones around her, and then dropping it before them. Birds do not appear to possess taste, probably fishes do not either, though as the smell of both is exquisite, and as the nasal and oral membranes are continuous, it is difficult to deny it to them.

The desire to acquire is an instinct exhibited by the babe in the cradle who tries to grasp at everything. It is also exhibited by the squirrel in the forest, who gathers nuts not only for present food but future use, and hides them in the hollow beech-tree, unknowing that winter will come and pass, when, after his long sleep, he will wake to starve unless a store be provided. And the child is as eager as the squirrel to gather and to keep. Acquisitiveness reasons not, neither does it perceive, it is excited into activity by the intellect, and should be controlled by it; rarely is it to be found acting alone, it runs in couples with secretiveness, which before we understand its nature we must describe.

Secretiveness, or the desire to keep, seems a propensity as innate in animals and man as is the desire to gain. Its tendency is to conceal. It is exceedingly developed in the wolf, the fox, spiders, and many others of the animal kingdom.

According to Dr. Pattison the Hindoos exhibit this propensity in a high degree in the form of cunning or duplicity. Wellington was famous for, and because of it; like Louis XI. of France, he is reported to have declared that if he thought a hair of his head knew his intentions, he would cut it off and wear a wig. As the abuse of this instinct is to lie, that is, to conceal the truth actively, so do very secretive people generally deceive. Wellington never did. He was one of the very few men who stood on the precipice but never fell over. His great rival, Napoleon, never scrupled to call black white whenever it suited his policy, though he was truthful enough in private life. Napoleon the Third is perhaps the most secretive man that ever lived, and probably truthful in his domestic relations; but men act in a corporate or political capacity in a manner that would scarcely be tolerated in private:—his whole political life has been a lie—a successful one if you will, but still a lie; and in our own Parliament men at times suppress the truth to serve a political purpose; and in courts of justice, where the truth, the whole truth, and nothing but the truth, should be worshipped, men are counselled to plead *not* guilty when they are guilty; witnesses are almost invited to perjure themselves by being asked if they are "for the plaintiff or defendant," and a counsel has been known to call God as witness to the innocence of a murderer, when at that very moment the confession of the man's guilt was in his pocket. The whole system of our law proceedings requires reform. John Doe and Richard Roe are declared to be creditors to some unfortunate, although there are no such people. Men are indicted for stealing one thousand pigs and five hundred sheep, when only one pig and one sheep are known to have been lost; and an attorney's bill of taxed costs means that he may charge as much more than the law allows as his cupidity may suggest. Secretiveness seems to permeate trade—false news, false assertions—goods in shop windows, marked with a large 1s. in ink, and a small 10d. in pencil, are practical lies, and should be punished as such; in fashionable society, the "not at home," the friendly greeting hiding a slanderous hate, the rouge on the face, and the falsehoods on the head, all evidence secretiveness as the agents, whatever other motive may have been the instigator—whether vanity or pride.

Women are more secretive than men. They are brought up to conceal their thoughts, and falsehood is too frequently resorted to as the surest means of doing so. Maid servants are very commonly driven by fear to shelter themselves behind it, for we expect them to be perfect even though we ourselves are not, and bitterly condemn the errors of humanity they exhibit. As a rule, the most truthful class of men I am acquainted with are agricultural labourers, and they are among the most independent.

Individual cases of an extraordinary development of this faculty are common enough.

Ann Ross was treated by Mr. Carmichael for what were afterwards found to be simulated insanity, epilepsy, asthma, and excruciating pains in the limbs, for which she underwent several operations with great fortitude, and afterwards had an arm taken off. The disease she had herself produced by thrusting needles into the limb, several of their points having been found, even in the bones. She was eminently pietistical, never without her Bible in her hand by day, and at night under her pillow.

I myself saw Mr. Luke at the London Hospital, cut down upon a needle, and extract it from the painfully sore leg of a girl, one of many needles so found, and which were inserted by herself.

One boy, a liar from his cradle, was sent to gaol, and the governor offered him a reward to abstain from lying for one week. He answered that he would not make the atttempt, *for he knew he should fail.*

I was consulted about a boy at Kidderminster, who would never give a direct reply to anything. When asked if he had been out or dined, or any other question, to which there could be no demur, about which no doubt could exist, he would say, "I think so," "I believe I have.' Never yes or no. Craniology disclosed secretiveness, conscientiousness, and fear, as dominant features in his mind. I asked if he had always been truthful, and was informed that he had been a liar from his cradle ! Having been convicted of his sin, he so feared now to commit himself, that he never risked the chance.

It is a prudential instinct, common to animals and man, capable of abuse in him, but not in them. The reason why will appear hereafter.

But it is in their combined action that these two instincts, acquisitiveness and secretiveness, play so important a rôle in the affairs of life. Under their influence men rise up early, and so late take rest, and eat the "bread of watchfulness and care," that they may gain and keep what their intellects may suggest—and it is well. But here steps in a moral law, that affects every mental power, but which, as far as I know, has been unnoticed by preceding writers, although its importance can scarcely be exaggerated. It is this, that whenever any of our faculties are used as ends instead of means, the object for which they were given is entirely defeated ; thus we should eat and drink to live ; so surely as we live to eat and drink, we die. Used as means the joint action of acquisitiveness and secretiveness produce industry, competence, and wealth. We are justified in earning our daily bread, in storing up food and raiment for that time when "the night cometh and no man may work." The comforts, and even luxuries of life, are legitimate objects of exertion. Our children, our friends, the hungry and the naked, the wounded and the sick, are all interested in our success, for they have all a right to share in it, and the education of the young, the sustenance of the old, and, above all, the spread of true religion, offer to the wealthy man opportunities which it will peril his soul to neglect, of "lending to the Lord." But so soon as man gains for the sake of gain, and keeps, because to hoard with him is his greatest pleasure, every object of these faculties is lost. The Nemesis of unholy wealth surrounds him. He becomes a contracted misery, a miser. He casts off his luxuries, denies himself his comforts, and is an enemy to his friends ; Midas like, he turns everything he touches into gold, and dies starved amidst the heap. "Oh, it is easy to be rich ! it is only to trust nobody, to help nobody, to get everything we can and save everything we get, to stint ourselves and everybody belonging to us, to be the friend of no man, and have no man for our friend ; to be mean, miserable, and despised, for some twenty or thirty years, and riches will come as surely as disease and death."* Remarkable instances abound in every

* Paulding.

country, have appeared in every age, illustrating the truth of this.

At Eckington, Worcestershire, not many years since, there died, at an advanced age, Mrs. Mary Barnes, on whose premises there were discovered after her decease 542 gown pieces, upwards of 100 gowns, and a large assortment of valuable shawls. She usually had in her house fourteen cats, and a great number of rabbits.

A female mendicant, about the same period, was taken before the magistrate of Amboise, France. On investigation, it appeared that she was the owner of a small cottage, for which she had paid 500f. A sum of 2600 francs in silver money, and 8000 francs in two-sou pieces, were found hid away in closets, chests, baskets, and wherever she could find a hole. She had ninety knives, and plate, linen, and jewellery in abundance, all of which she had earned by gathering herbs and collecting manure from the roads. Queen Elizabeth is said to have had at her death upwards of 1000 dresses, and other goods in proportion.

The history of old Elwes the miser is well known. How he gathered bones and eggshells on the high road, for broth, and yet could give his thousands when the whim took him. But as remarkable a man as any was old Daniel Dancer, of Harrow Weald. It is reported of him that he lived a life of abject want. He allowed his sister and himself one red herring and one hard dumpling each for dinner; he never burnt a candle, but sometimes indulged in a rushlight, which he thus obtained. He carried a snuff-box, and begged a pinch of all he met, which he put quietly into his box. When full he exchanged it with the grocer for a rushlight. He wore haybands for stockings, and went al fresco whilst his one shirt dried. He was subpœnaed once to a trial at Aylesbury. He rode thither with a friend. On his arrival he told his friend to order for himself and horse whatsoever he would; he did so, and the charge was 15s., which Daniel cheerfully paid. His own expenses amounted to twopence for some bread and cheese, which he eat in the stable with his horse, shared the water in his pail, and slept under the manger. He died worth 3000*l.* per annum. In stockings and chairs, in garrets and haylofts, up thatch and chimney, guineas, golden guineas, tumbled out by thousands; and his misery was not the result of early poverty, as is sometimes the case, where much has been suffered and much is feared, but the result of two of our strongest propensities, used as an end, instead of a means, and illustrating the moral law that governs us.

Man is omnivorous; in common with the carnivoræ he has the power and the disposition to destroy, and rightly used destructiveness is as proper to his nature as is benevolence.

That the propensity is innate, may be easily seen in children, who in early infancy are more cruel than cats; that it is more intensely manifest in the carnivoræ than the herbivoræ is also evident, but the latter have it. The worm even will turn if trod upon, and the poor timid hare will bite the hand that grasps it, and fight to the death when it meets a rival in the field. That the instinct is dependent upon the brain and not the stomach, has been well observed by Gall, who says, "Give the teeth and claws of a tiger to a sheep, and you do not therewith transfer the sanguinary disposition. It is not therefore hunger or thirst which induces to slay. And they who examine the stomach or teeth of an animal to determine its class, are examining but instruments, in harmony it is true with the disposition, but not creating it. He calls it "L'instinct carnassier, penchant au meurtre." But this is a mistake which Spurzheim corrected. He names it the desire to destroy, which appears to be a perfect definition. Nothing, however, more clearly proves Dr. Gall's honesty, than this and other mistakes. He found murderers, such as Gottefried, exhibiting a peculiar cranial conformation, and he named the motive power after its abuse, not its use. Beasts do not murder, they merely destroy, and yet they have it. Gottefried was executed for murdering thirteen persons, viz., both parents, two husbands, four children, her brother, and several acquaintances. Louis XI. of France was at heart a tiger. When James D'Armagnac was executed for treason, Louis caused his sons to be placed under the scaffold that *their father's blood might fall upon their heads*. Like our own Richard, he could smile, and murder whilst he smiled. He caused four thousand persons to be executed during his reign. That Chinese devil, Yeh, is credibly supposed to have executed more that seventy thousand.

It may be said that these latter atrocities were committed under a cold-blooded policy, without any desire to destroy, only to remove, and might be equally perpetrated by the gentle and kind, as by the naturally savage man. Granted. Some of the most religious as well as most amiable men, have caused their thousands to be slain. Butchers need not be, and as a rule are not, cruel, but some are, and there is a wide difference between those who, like our own judges, destroy life as a duty, and those who delight in blood and gloat over the sufferings of others. It is when excited that all the feelings stand prominently forward, clear of the ranks, and free from all intermixture—it is then that they can be analysed and defined, and from such evidence we determine destructiveness to be an innate primitive instinct, and not an intellectual faculty. The instinctive desire to defend oneself is not a modification of the desire to offend another.

The bravest among all animals, including man, are generally the quietest, and we distrust the courage of a passionate man, as much as we do his judgment. Timid animals hiss and bark, bold ones meet the danger as pious men pray, silently:

"Now shrieked the timid, and stood still the brave."

"When defending one's life," said Major Murray, who preserved his against fearful odds in Northumberland Street, Strand, "one must not waste one's breath;" bullies endeavour to intimidate the enemy they know they dare not oppose. But man may possess abundance of both bravery and cruelty and be like the tiger, or he may have little of either and yield to every foe. But there are two kinds of courage, the one physical so called, the other moral; the one faces danger impulsively without considering means or ends, as Charles the Twelfth of Sweden did when he turned sharply round upon the burning shell that fell into his tent, and moral courage was as strikingly exhibited by him, when folding his arms he stood undaunted and erect until it exploded. His Secretary displayed neither, for he jumped out of the window. Metaphysicians, and especially moralists, have been altogether lost in their estimates of these two faculties. By some it has been considered that a man may be brave if he will, cowardice has been looked upon, not as a weakness but a crime, and to Gall we owe the true description of its nature.

The North American Indian is more firm than combative; he counts the odds, attacks in the dark, and slays the defenceless. But take him prisoner, let the pride of his tribe be bound up in his person to the stake, and let his foes torment him with all the ingenuity of devils, he suffers in silence; "for the son of Aornooko will never complain."

I have said that physical courage is excited into activity by danger, it operates at once and without reflection, animals have it to an extent as great or greater than man; but moral courage they have not; it is called into play by reflection, and is the result of calculation; but thousands calculate and yet are not firm. Dr. Gall calls combativeness "Instinct de la defense de soi-même, et de sa propriété: penchant aux rixes, courage,"* and gives several illustrations in support of these definitions. He gathered together a variety of characters, gave them money and wine, and obtained their confidence. He then noted their habits, manners, and conversation, and separated the bold from the timid; this he did for craniological purposes, with which at present we have nothing to do, but it is necessary to advert to his mode of investigation, and reasons for it, in order to judge of the value of them. "Dans

* *Sur les Fonctions du Cerveau*, tome iv. p. 1.

certain cas," he says, "il est bien plus facile de découvrir l'organe qui determine une certaine manière d'agir, que la qualité ou la faculté fondementale elle-même. Des actions qui sont une suite de l'activité extraordinaire d'un organe, frappe beaucoup plus que la destination primitive de cet organe, et sa manière d'agir ordinaire. C'est par cette raison que j'ai été dans le cas de commencer par observer presques tous les organes, toutes les qualités, et toutes les facultés dans leur activité excessive. Lorsque les qualités et les facultés sont une fois reconnues comme propres et indépendantes, il est possible d'en inferer peu-à-peu la destination primitive d'un organe."* Well, he found some of these men peaceable, some quarrelsome, and erroneously inferring courage from violence, he taught that love of quarrelling was an excited manifestation of combativeness,—I have already shown that it is just the reverse. But he instances also one of his friends as having been expelled from several universities as a duellist, and of another whose delight it was to excite the revellers at a wine shop, put out the lights, break the furniture, and fight pellmell in the dark, with chairs or anything that came to hand. He knew a young girl, who from her infancy was accustomed to dress herself in boy's clothes, and mixing with the gamins in the street, very soon became the hero of a fray; she continued the habit after she was married. But his chief argument is derived from its supposed diseased manifestations in insanity, and he cites several cases from Pinel of great violence both of word and deed in proof. One, of a religieuse at the Hospital Salpêtrière, who heaped upon all around her every possible outrage and abuse; swearing at, and attempting to strike every one who approached her, tearing her clothes to pieces, and remaining naked. "She dared not brave the authority of the Governor when he was present, but abused him behind his back." Another, an only son, humoured and spoilt, gave himself up to every caprice, attacking with audacity all who opposed him. Let any animal offend him and he instantly put it to death. He generally involved himself in a fight in any assembly he joined, and left it bleeding; he was confined at length in the hospital of Bicêtre.

Although he declares it to be very difficult to discover the fundamental force of a faculty, yet he believes that "the instinctive desire for quarrels and combats may be traced in all its ramifications to the instinct of self-defence or combativeness. And both Spurzheim and Combe endorse and adopt his opinion. But the very essence of combativeness is "self-defence" not offence, and no exaggerated manifestation of it can alter its character; destructiveness is the instinct of offence, and who does not recog-

---

* Ibid. pp. 1, 2, *et seq.*

nise at once in the cases quoted its legitimate although diseased action? And one is astonished how Gall could have fallen into so grave an error, and still more so that it should have remained undiscovered until now. But a word and a blow, and frequently the blow first, are so commonly associated, and the courageous man is always so ready to attack if no other defence of himself succeeds, that at first blush Gall would seem to be correct.

But violence, whether of tongue or hand, is the essential attribute of destructiveness; from the sneer to the stab, from malice to murder, the gradations are short and sure, the links are perfectly sound and coherent, and insanity only rivets them the faster. Strange that Gall should not have seen this—still more strange that he should have witnessed *the violence committed by lunatics upon themselves, and attributed it all to self-defence.* But how is it that if a brave man be attacked, he very soon quits his defensive for an offensive attitude? I reply that a sympathetic action connects the whole of the separate mental powers together, that the excitement of one very soon engages others, and that combativeness cannot be aroused in any man without very soon bringing whatever amount of destructiveness he may possess into play. "Stand back!" he exclaims to his assailant, "stand back, I don't want to hurt you, but shall not put up with any nonsense." Well, but the opponent wont stand back; he abuses, he strikes, and the brave man wards off the one and treats with contempt the other, till at last finding his defensive attitude useless, he steps forward and knocks the bully down, with some such remark as, "Well, you would have it, and now you've got it! Another time you'll leave peaceable men alone." In fact, the two instincts are very good friends, and very ready to assist each other. A bold savage attacks openly, a timid one cunningly. The combined action of one or more of the mental powers constitutes the various characters observable among men. And when we know that they vary among themselves both in strength and activity, the actions of men are as many motived and as interminable as are the changes from a peal of many bells. Moral courage has been supposed to be but another term for firmness, which Combe denies to animals because they have no moral sentiments, and because it has always attracted the attention of psychologists, when exhibited in defence of some moral principle; but it is a misnomer altogether, it is as strikingly displayed by the murderer as the martyr. Thurtell, who was executed at Hertford for the murder of Weare, one of the most cold-blooded upon record, exclaimed, "Now I will show them how a brave man can die!" and neither blanched in cheek nor shook in limb. It is still more flagrantly wrong to call it firmness, and deny it to animals. I think it is clearly demonstrable that there is no such a primitive faculty as firmness, consequently no organ of the brain to manifest it; and up to this time I have erred, as well as all other phrenologists, in believing that there was.

How is it attempted to be proved? listen to Gall! He says that "the character of man depends much more upon the sentiments than the faculties. A feeble and indecisive man and a man of firm character are equally ignorant; the first, because he floats from one project to another; the second, because he persists determinately in the path he has taken:"* and he cites Cicero as an instance of wavering uncertainty, and Cato of fixedness of purpose. Of the latter, he relates that Pompedius demanded of him, when a child, that he should be reconciled to his uncle. The boy was silent, but showed by a look and an air of dissatisfaction that he wished not to do what was demanded of him. Pompedius insisted, and taking him in his arms, carried him to the window, threatening to throw him out if he persisted in his refusal. But fear had no effect upon him. He would die rather than submit to his enemy.† Children generally may be divided into two classes—those who yield, and those who do not. Men differ in a similar manner. The vacillating man is not to be depended upon, either by himself or others; the unswerving man is to be depended upon by all. "Tu ne cede malis," is his device; menaces and danger only make him more firm; of such it may be said, "Si fractus illabatur orbis, impavidum ferient ruinæ." "Take any form but that, and my firm nerves shall never tremble," says Shakespeare; and Scott illustrates it in the character of James Fitz-James—

"Come one, come all, this rock shall fly
From its firm basis, soon as I."

But who does not perceive that in all these and similar instances there have been intellectual faculties at work, and many sentiments aroused. Conscientiousness, love of approbation, pride, courage, with many more, are excited and combined, and each for itself will never yield.

The lower animal instincts are equally obstinate. Children will look you in the face and lie, and no punishment will sometimes elicit the truth. I knew a girl who stole some small thing, and when taxed with it, and even promised forgiveness, declared and persisted that she had never seen it, although it was in her clenched hand all the time. Animals are even more firm than men. Try to take a horse from a stable on fire, and he will die first. The obstinacy of fear is scarcely excelled by that of conscientiousness. Self-esteem seldom gives up—veneration rarely

---

* Tome v., pp. 399, 400, *et seq.*

—ignorance never; and the firmness of a brute always exceeds that of man. Try to drive a pig, try to urge a donkey or a mule the way he would not go; with what firmness a bull-dog keeps his hold, how pertinacious in his attempts to escape is Edmond's hyæna, how consistent, how certain, are all the actions of animals, and yet forsooth they are said to have no firmness! The truth is, that there are two kinds of firmness, as there are of courage—the one consists in an excitement and persistent action of any of the lower faculties, and is common to animals and man; the other is the result of thought, and involves principle; it is the determination of the whole mind upon the whole matter, and should be called will, only that animals have a will, but have not this. Moral courage is not its proper designation, because a man's social and selfish feelings have all a voice in the matter as well as his moral sentiments; a mother's love for her child will produce an amount of firmness not to be quelled by man; decision is a better term, because it presupposes nothing, but describes the fact. The firmest men are those nearest allied to the brute, they are firm by impulse, and it is oftener called into play; fear is the only thing that checks them, and that of course only in proportion to its amount.

The most vacillating men are those endowed with the largest intellect and the higher moral sentiments. They must canvass a proposition in all its bearings before they decide, and consult the divinities of their nature before they act. But this once done and their minds made up, "quod si cœlum ruat," they are immoveable as the earth. The firmness of an Indian arises from his pride, of a religious man from his creed, of a conscientious man from his sense of right, but these are all antagonized, and in proportion to his doubt is the wise man's indecision. Lord Eldon doubted, and decided not; Lord Brougham has wandered through every phase of science—I had almost said of politics. But the merely animal man, the unreflecting man, wavers not. George the Third was an instance of the latter, thousands upon thousands of the former. Cranmer only evinced firmness in his death, he had been lamentably deficient of it until then. Women are more firm than men, first, because they are more secretive, and secondly, because they have been hitherto less cultivated. Men who have mastered any subject whatever, who have confidence in themselves, and who are not swayed by vanity, nor passion, nor prejudice, nor fear, are firm as rocks. Wellington was one of these, and perhaps Napoleon III. is another.

Fear has been well called the master passion of the mind. It is common to all sentient beings, but very differently manifested in each: timid as a dove or a hare is a household phrase, bold as a lion is equally so, but the absence of fear does not produce courage, though it contributes to presence of mind, and the absence of courage does not produce fear, though it leaves it unantagonized. Gall attributes to fear properties belonging to secretiveness and conscientiousness; he calls it "circonspection, prevoyance," and cites two illustrations; the one a prelate who hesitated so much that it was painful to listen to him, the other a councillor of state, who, from his eternal irresolutions, obtained the name of Cacadubio. And Combe, following up the idea, has named it cautiousness. That it is a primitive faculty there can be no doubt; if there be a single mental manifestation reduced to its root this is the one. It is entirely independent of intellect, or of health, or age, or sex. It varies in amount in animals of the same species almost as greatly as in different races. It has dwelt in palaces surrounded with guards, and been almost absent on the rocking mast where the sea-boy slept. It has hunted a Louis XI., a James I., an Aristippus, and a Paul, from chamber to chamber in the vain hope of repose, and allows not the miser to rest away from his hoard. It is not judgment nor prudence nor foresight, still less is it caution, though it may produce it. To fear coming evil just enough to arrest the career and enlist the intellect of those who are rushing headlong into it, allows other instincts to come into play. But when excited beyond this, when obeyed as the end instead of the means, fear produces the greatest possible imprudence and incautiousness.

Under its unbridled influence the suicide "rushes into the arms of death to avoid the terrors of his countenance."* Shrieking women and timorous men snatch the reins from the driver's hand when the horse runs off, upset lurching boats by rushing to the toppling side, "et corde et genibus trement" before the infuriated bull, and fall headlong from the cliff a bold man folds his arms upon in safety.

If fear and cautiousness were synonyms, the increase of one would be the increase of the other, whereas, beyond a certain point, where fear shakes itself loose from its neighbours, it paralyses and destroys.

These constitute as far as I yet know the selfish propensities common to animals as well as man. They are given for his self-preservation, and are as capable of their fullest action in the wilderness, away from all human beings, as in the crowded haunts of men. Robinson Crusoe exhibited them as well before Friday joined him as afterwards. He ate and drank, destroyed fish and

---

* Young.

birds and beasts for food. He acquired and secreted with a squirrel's instinct and with human caution. He trembled at the print of the savage foot in the sand, then shot its owner; and he exhibited as much moral courage as physical during his whole career.

Except in monomania these instincts rarely act alone, and even then their independence is of short duration. But it is curious to witness the result of their combined action, and to trace the several motives by which an animal is impelled. I remember walking in Belfast when an urchin from a hovel threw a stone at two dogs leisurely walking on before me. The one was a bull-dog, the other a spaniel. The stone passed between them. The spaniel yelped and fled, the bull-dog turned short round on his hind legs as on a pivot, and seeing no one but me (for the boy had concealed himself), uttered a most menacing growl, as much as to say, "Don't do that again ! my combativeness is always up, my destructiveness is easily aroused." I stopped short, and almost cried, "It wasn't I," but I was silent, and after another growl he turned and went on his way, only more slowly than before. The spaniel ran some twenty yards and stopped, then taking courage from his friend came rushing back open-mouthed and noisy, and would have torn me to pieces if he had dared. The animals went their way, the bull-dog every now and then checking my advance by a retrospective look and snarl, until they reached their turning home, when he stopped, and looked, and growled again, then left me in peace. There are other instincts common to animals and man, but they come not within this category.

Before, however, parting from these, one or two points require notice. The retributive law already noticed attends their abuse. Combative men provoke offence, and are not always strong enough to defend themselves. A destructive man, whose hand is raised against every one, will find every one's hand raised against him ; and "whoso would save his life shall lose it." There is nothing so fatal as terror ; and the obstinate man is always kicked to the wall by those who have the power to do it.

If the above faculties constituted a man's whole affective mind, he would be a serpent. The devil is a personification of them ; he goes about seeking whom he may devour. He seeks to acquire the souls of men, he is the father of lies, his obstinacy is indomitable, he defies the Omnipotent, but he believes and trembles.

I have said that animals cannot abuse these or any other of their mental powers. But it would appear that the cat does when she torments a mouse. Unnecessary pain seems not to emanate from Divine beneficence, and unnecessary pain is uniformly inflicted by the cat. But I apprehend that man does this. It may almost be said of him, "non tetigit quod non damnavit;" and to its domestication may the cat's apparent cruelty be traced. In the wilderness the animal destroys for food, but in our houses we feed it, even to repletion, and although this tames much, it leaves destructiveness a leading and unsatisfied instinct, which now and then delights to enjoy itself. So when a cat catches a mouse, as it is not hungry, it just gives the prey a loving bite and lets it go, secretly watching from the corner of the eye, and springing upon it with a joyous bound when just about to escape, *the animal has caught the mouse a second time, which is, with it, equivalent to a second mouse*, and so enjoyment is prolonged and multiplied by the captive's agonies. The butcher indulges his acquisitiveness in a similar manner, when he tosses the hansel shilling in the air and catches it in its fall.

The miser hides his money that he may reacquire it every time he steals to his chest in the dead of the night, and opening the lid looks down with joy upon the treasure which every day and every night is lost and found, arousing modified feelings such as would in a greater intensity hail the advent of a really new and sudden acquisition of wealth. These instincts exist in different degrees in the same individual. A man may be timid yet brave, ferocious yet benevolent, truthful yet deceitful, but the intellect and higher feelings direct and develop these emotions, or are directed by them. We defer, however, any further remarks upon this point.

---

